# Hematologic Indices and Global Registry of Acute Coronary Events (GRACE) Risk Score in Acute Coronary Syndrome Patients in Ethiopia

**DOI:** 10.4314/ejhs.v34i6.6

**Published:** 2024-11

**Authors:** Samuel Tadesse, Esayas Kebede Gudina, Daniel Yilma, Elsah Tegene, Tilahun Yemane, Andualem Mossie

**Affiliations:** 1 Department of Biomedical Sciences, Jimma University, Ethiopia; 2 Department of Internal Medicine, Jimma University, Ethiopia; 3 Department of Medical Laboratory, Jimma University, Ethiopia

**Keywords:** Acute coronary syndrome, GRACE risk score, hematological indices, ACS prognosis, mortality risk, Jimma Medical Center

## Abstract

**Background:**

Prognostic ratings are essential for making quick clinical decisions. In patients with acute coronary syndrome (ACS), the Global Registry of Acute Coronary Events (GRACE) score is often used to predict in-hospital mortality. Hematological indices are strongly correlated with the likelihood of adverse outcomes in ACS patients, given the systemic hypoxemia and inflammation linked to its pathophysiology. This study aimed to assess the relationship between hematologic indices and the GRACE risk score in ACS patients.

**Methods:**

We consecutively recruited patients diagnosed with ACS at Jimma Medical Center (JMC) from May 1, 2022, to October 31, 2023. We performed biochemical analyses and complete blood counts, calculating GRACE scores. We correlated two continuous parameters and evaluated GRACE risk score independent predictors using multivariate linear regression analysis.

**Results:**

A total of 110 patients were included, with 74 (67.3%) being men. The mean age was 56 (±11) years. Significant correlations were found between red cell distribution width (RDW), mean platelet volume (MPV), plateletcrit, and platelet count with worse GRACE risk scores (r = 0.569, P <r = 0.585, P < 0.001; r = 0.400, P < 0.001; r = 0.274, P < 0.013, respectively). In multivariable linear regression, higher RDW and MPV were associated with higher GRACE risk scores.

**Conclusion:**

This study revealed significant differences in hematologic parameters among ACS patients with varying GRACE risk scores. Increased RDW and MPV were identified as independent predictors for high GRACE risk scores.

## Introduction

Accurate risk stratification of patients with acute coronary syndrome (ACS) is crucial for effectively targeting evidence-based therapies. It also helps identify high-risk patients who may benefit from advanced treatments. Numerous biomarkers and risk scores have been evaluated for this purpose(1). The Global Registry of Acute Coronary Events (GRACE) risk score is a validated tool for stratifying ACS patients according to risk and guiding treatment decisions(2). It incorporates factors such as heart rate, systolic blood pressure, serum creatinine, and serum troponin(3). However, the GRACE system focuses primarily on specific pathophysiological aspects linked to ACS outcomes. Additional information from biomarkers targeting different parts of the ACS pathophysiology can further guide treatment decisions.

Hematological parameters—including white blood cell count (WBC), neutrophil to lymphocyte ratio (NLR), platelet to lymphocyte ratio (PLR), mean platelet volume (MPV), red cell distribution width (RDW), and WBC to MPV ratio (WMR)— can provide valuable insights into prognosis, risk assessment, and optimal treatment for ACS patients(4). Recent large-scale studies have shown that hematologic markers are strong predictors of cardiovascular events in individuals with coronary artery disease(5-7).

In clinical practice, RDW is commonly used as part of routine complete blood counts (CBC) to differentiate among types of anemia. Higher RDW is associated with mortality in both the general population and in patients with heart failure, acute coronary syndrome, and stable coronary artery disease(8). Factors influencing RDW in ACS include inflammatory stress, adrenergic activation, neuro-hormonal pathways, and dietary deficiencies(9).

Platelets play a critical role in the pathophysiology of ACS(10). MPV is an easily measurable biomarker of platelet activity. Elevated MPV has been linked to known cardiovascular risk factors such as dyslipidemia, hypertension, diabetes, smoking, and obesity(11, 12). Compared to patients with stable angina, those with ACS exhibit higher MPV levels(13). Research has shown that MPV can predict reperfusion damage, recurrent myocardial infarction, mortality, and restenosis after percutaneous coronary intervention(14, 15).

While studies in developed countries have explored the relationship between hematological indices and the GRACE risk score, there is a need for more research in sub-Saharan Africa, where infectious agents can impact hematologic biomarkers. This study aims to provide baseline data to improve the management of hematologic markers in diagnosing, assessing risk, and following up with ACS patients, especially in resource-limited settings like Ethiopia.

## Patients and Methods

**Research design and data collection**: This prospective cross-sectional study involved consecutively recruited patients diagnosed with ACS at Jimma Medical Center (IMC) between May 1, 2022, and October 31, 2023. We monitored patients to evaluate the role of hematologic markers in risk classification until discharge or death after baseline data collection within 24 hours of admission. Each patient underwent a complete physical examination and assessment of coronary risk factors, with clinical symptoms recorded. Prognosis was evaluated based on Killip clinical examination standards.

Patients under 18 years old, those with hematological disorders, known malignancies, systemic inflammatory or autoimmune diseases, chronic liver disease, renal failure, or those on immunosuppressive therapy, anticoagulants, or readmitted were excluded. Participants were subjected to face-to-face interviews and laboratory investigations. We categorized patients into three groups based on computed GRACE risk scores: low (less than 109 points), intermediate (109-140 points), and high (more than 140 points). Previous studies have shown that the GRACE risk score outperforms existing ACS risk scores in terms of discriminative performance(16, 17).

**Laboratory analysis**: Upon admission, a venous blood sample was collected under aseptic conditions. Trained laboratory personnel performed complete blood counts and biochemical analyses. CBCs included hemoglobin, RBC count, RDW, WBC count, and differential counts (neutrophils, lymphocytes, eosinophils, basophils, and monocytes). We analyzed platelet counts and MPV using the Uni-CelDxH 800 Coulter Cellular Analysis System. Calculations were made for NLR PLR, MPVLR, and WBC to MPV ratio. Relevant serum biochemical measurements, including high-sensitive troponin I and serum creatinine, were measured using a Roche Cobas Integra 400 analyzer. CBC results were available within 30 minutes, while serum chemical analyses took one hour. We ensured sample quality and analysis adhered to standard operating procedures.

**Data management and analysis**: We performed statistical analysis using Stata-SE version 14. Categorical variables were described using frequencies and percentages. We used chi-squared and Fisher's exact tests to assess associations between categorical variables. The Kolmogorov-Smirnov test checked for normal distribution in continuous variables, which were expressed as mean ± standard deviation. We used one-way analysis of variance and the Kruskal-Wallis test as appropriate for group comparisons. The Spearman correlation coefficient calculated correlations between continuous parameters. Multivariable linear regression analysis assessed independent predictors of GRACE risk scores. A P-value of <0.05 was deemed statistically significant.

**Ethical considerations**: The Jimma University Institutional Research Board approved the study protocol (IHRPGD/554/2022) following the Declaration of Helsinki. All study participants were informed about the study's purpose and provided written informed consent.

## Results

**Socio-demographic and clinical characteristics of ACS patients**: A total of 110 ACS patients were included, with 74 (67.3%) being men. The mean age was 56.7 ±11.9 years. STEMI was diagnosed in 99 (90%) patients, and 90 (81.8%) were discharged with improvement. Hospital stays ranged from 6 to 46 days, averaging 14.5 ± 6.7 days. According to GRACE risk scores, 28 (25.5%) patients were classified as low risk, 45 (40.9%) as intermediate risk, and 37 (33.6%) as high risk. More than half of in-hospital deaths (n = 11, 55%) occurred in the high GRACE risk score group. The mean GRACE death probability score for those who passed away in the hospital was 29% ± 12.1. Table 1 presents the demographic features of patients grouped by GRACE risk scores.

**Table 1:** Demographic and clinical characteristics of ACS patients by the GRACE risk score group

Variables		Low GRACE Risk Score	Intermediate GRACE Risk Score	High GRACE Risk Score	p-Value
Age, years (Mean ±SD)		46.1±8.7	56.5±9.4	64.8±10.4	0.186
Sex	Male, n (%)	18(18.8)	27 (30.3)	29 (24.9)	0.913
	Female	10(10.9)	18(17.6)	15(14.5)	
Hypertension, n (%)		12(11.0)	22 (20.5)	16 (18.5)	0.426
Diabetes Mellitus, n (%)		4 (7.2)	14(13.6)	15 (12.2)	0.147
Family history of ACS, n (%)		2(3.3)	3 (5.3)	8 (4.4)	0.076
STEMI* n (%)		25 (25.2)	37 (40.5)	37 (33.3)	0.004
Severe Angina, n (%)		28 (22.4)	37 (36.0)	23 (29.6)	0.001
Latency (> 4 hours), n (%)		28 (27.2)	45 (43.8)	34 (36.0)	0.048
Patient outcome;	Deceased, n (%)	2(7.1)	7(15.6)	11 (29.7)	0.054
	Discharged	26(92.9)	38(84.4)	26(70.3)	
GRACE† probability of death (%)		3.34±2.l	9.27±2.8	29±12.0	0.001

* ST-elevation myocardial infarction

† Global registry of acute coronary event

**Hematological and biochemical characteristics of ACS patients**: Among hematological indices and biochemical markers, higher mean values of RDW, neutrophil count, eosinophil count, NLR PLR, MPVLR, plateletcrit, high-sensitive troponin I, and serum creatinine were observed in patients with worse GRACE risk scores. Conversely, MCH, lymphocyte count, and basophil count were lower in this group (Table 2).

**Table 2:** Hematological and Biochemical characteristics of ACS patients by the GRACE risk score group

Variables	Low GRACE Risk Score	Intermediate GRACE Risk Score	High GRACE Risk Score	p-Value
White blood cell count 10 ^3^/µl, (Mean ±SD)	10.47±4.3	10.9±4.1	10.9±4.3	0.105
Red blood cell count 10 ^6^/µl ,(Mean ±SD)	4.48±0.8	4.9±0.9	4.8±l.5	0.360
Hemoglobin gm/dl, (Mean ±SD)	13.1±2.8	14.1±2.5	13.1±2.8	0.373
Hematocrit- % (Mean ±SD)	39.0±7.7	41.9±6.7	40.3±8.6	0.479
Mean corpuscular volume fl, (Mean ±SD)	87.77±9.2	87.8±6.6	87.6±8.3	0.139
MCH*pg, (Mean ±SD)	29.26±3.5	29.4±3.1	28.6±2.3	0.005
MCHC†mg/dl, (Mean ±SD)	33.1±2.6	33.3±2.6	32.5±1.9	0.135
RDW-CV‡% (Mean ±SD)	17.3±1.2	19.3±1.6	21.3±1.4	0.001
RDW-SD fl, (Mean ±SD)	55.0±3.8	61.4±4.9	67.6±4.6	0.001
Platelet count 10 ^3^ /µl, (Mean ±SD)	182.6±71.5	221.8±69.2	265.6±105.5	0.081
Mean platelet volume fl, (Mean ±SD)	9.9±0.84	11.8±1.1	12.7±0.6	0.069
Neutrophil count 10 ^3^/µl (Mean ±SD)	7.57±3.5	8.9±4.2	8.9±3.8	0.027
Lymphocyte count 10 ^3^/µl (Mean ±SD)	1.46±0.85	1.2±0.5	1.04±0.4	0.009
Monocyte count 10 ^3^/µl (Mean ±SD)	0.69±0.4	0.65±0.4	0.82±0.59	0.170
Eosinophil count 10 ^3^/µl (Mean ±SD)	0.17±0.2	0.12±0.13	0.18±0.2	0.001
Basophil count 10 ^3^/µl (Mean ±SD)	0.14±0.2	0.44±0.07	0.08±0.15	0.001
Neutrophil to lymphocyte ratio (Mean ±SD)	7.78±11.3	11.8±15.87	10.4±10.3	0.001
Platelet to lymphocyte ratio (Mean ±SD)	177.5±166.5	262.7±287.9	299.9±204.7	0.001
WBC to MPV ratio (Mean ±SD)	1.04±0.4	0.94±0.37	0.86±0.34	0.213
MPV to lymphocyte ratio (Mean ±SD)	9.65±7.7	14.7±16.46	14.3±7.4	0.001
Plateletcrit- % (Mean ±SD)	0.0002±0.00008	0.00025±0.00008	0.0003±0.0001	0.027
High sensitive troponin-I, µg/dl (Mean±SD)	43.4±19.5	76.0±39.8	139.3±39.5	0.001
Creatinine, mg/dl Mean (SD)	0.88±0.28	1.35±0.44	2.68±1.06	0.001

* Mean Corpuscular Hemoglobin

† mean corpuscular hemoglobin concentration

‡ red cell distribution width

**Relationship of hematological indices with GRACE risk scores of ACS patients**: Spearman correlation analysis indicated a positive moderate correlation between higher GRACE risk scores and RDW-CV (r = 0.5659; p < 0.001), RDW-SD (r = 0.5699; p < 0.001), platelet count (r = 0.2742; p < 0.013), MPV (r = 0.5853; p < 0.001), and plateletcrit (r = 0.4004; p < 0.001) (Table 3). We reviewed potential variables for the final model using univariable regression analysis, finding that age, platelet count, RDW-CV, and MPV were potential predictors of GRACE risk scores. Multivariable linear regression analysis confirmed that high RDW and MPV were independent predictors of the GRACE risk score. A unit increase in RDW-CV % and MPV resulted in a 0.326 and 0.262 rise in GRACE risk scores, respectively (Table 4).

**Table 3:** Correlations of haematological indices and their derivatives with GRACE risk score

Hematologic Indices	GRACE risk score	
	r	P-Value
White blood cell count 10 ^3^/µl	0.1039	0.3562
Red blood cell count 10 ^6^/µl	0.1787	0.1104
Hemoglobin gm/dl)	0.2050	0.0664
Hematocrit- %	0.2075	0.0631
Mean corpuscular volume fl	-0.0274	0.8085
MCH* pg	0.0326	0.7724
MCHC† mg/dl	0.0102	0.9283
RDW-CV‡ %	0.5659	0.0001
RDW-SD fl	0.5699	0.0001
Platelet count 10 ^3^/µl	0.2742	0.0132
Mean platelet volume fl	0.5853	0.0001
Neutrophil count 10 ^3^/µl	0.1257	0.2635
Lymphocyte count 10 ^3^/µl	-0.1646	0.1420
Monocyte count 10 ^3^ /µl	0.1126	0.3167
Eosinophil count 10 ^3^/µl	-0.0497	0.6596
Basophil count 10 ^3^/µl	-0.1922	0.0856
Neutrophil to lymphocyte ratio	0.0768	0.4958
Platelet to lymphocyte ratio	0.1738	0.1207
WBC to MPV ratio	-0.0781	0.4880
MPV to lymphocyte ratio	0.1152	0.3056
Plateletcrit- %	0.4004	0.0002

* Mean Corpuscular Hemoglobin

† mean corpuscular hemoglobin concentration

‡ red cell distribution width

**Table 4:** Multivariable linear regression analysis of predictors for GRACE risk score

Variables	Unstandardized Coefficients		Standardized Coefficients				
						
	Coef.	Std. Err.	β	t	P value	95% Confidence	Interval for β
AGE	1.037	.210	.385	4.92	0.001	.6175773	1.45732
RDW CV* %	5.078	1.448	.326	3.51	0.001	2.193348	7.964416
Platelet	.0329	.025	.101	1.30	0.197	-.017466	.0834059
MPV†	5.424	1.997	.262	2.72	0.008	1.446349	9.401879
Constant	-104.08	23.502		-4.43	0.001	-150.8931	-57.27502

* Red cell distribution width

† mean platelet volume

## Discussion

This study aimed to examine the relationship between hematological markers and the GRACE risk classification for ACS. We found significant differences in hematologic parameters among ACS patients with varying GRACE risk scores. RDW, MPV, plateletcrit, and platelet count were significantly correlated with worse GRACE risk scores. Furthermore, multivariable linear regression analysis indicated that RDW and MPV were independent predictors for higher GRACE risk scores. The inflammatory processes underlying atherosclerosis contribute significantly to plaque disruption, potentially leading to thrombus formation and ACS(18).

The study found that 28 (25.5%), 45 (40.9%), and 37 (33.6%) patients were classified as having low, intermediate, and high GRACE risk scores, respectively. Aging correlated with higher GRACE risk ratings, with the mean age of participants being 56.69 ± 11.91 years and a predominance of males (74, 67.3%). This outcome aligns with findings from the Makassar study(19). However, Dai et al. reported that around 60% of ACS patients admitted were over 65 years old, suggesting a possible shift in disease patterns in African countries(20). Compared to Europe, higher rates of younger individuals with non-infectious diseases, particularly heart disease, have been observed in Africa(21).

Our laboratory examination revealed that increased MCH, RDW, neutrophils, lymphocytes, eosinophils, basophils, NLR, PLR, MPVLR, and plateletcrit were significantly associated with elevated GRACE risk scores. Consistent with our findings, Oncel et al. found NLR linked to worse in-hospital outcomes in STEMI patients, independent of GRACE risk scores(22). Additionally, Dabbah et al. demonstrated a graded association between increasing RDW and mortality following acute myocardial infarction(23).

Correlation analysis showed a substantial positive correlation between GRACE risk scores and RDW, MPV, and plateletcrit. Multivariable linear regression confirmed that elevated RDW and MPV were independently associated with higher GRACE risk scores. Previous studies corroborate these findings, suggesting RDW can be used for short-term risk stratification in ACS patients. Moreover, combining RDW with the GRACE risk score enhances prognostic value for all-cause mortality(24-26).

Previous research has also established a positive correlation between MPV and GRACE risk score, indicating MPV could supplement the scoring system in predicting cardiovascular events in ACS patients(27, 28). Higher MPV levels are associated with increased thrombogenicity due to platelet enzymatic and metabolic activities(29). Conversely, some studies report lower MPV levels in ACS patients compared to those with stable coronary artery disease. Basic indicators like MPV and RDW can be beneficial in assessing mortality risk following ACS(7, 30).

This study is significant as it is the first in Ethiopia to explore the relationship between hematologic indices and risk stratification scores in ACS patients. A limitation is that participants were only observed during their hospital stay, and unexamined levels of erythropoietin, iron, folic acid, and vitamin B_12_ were potential confounders.

In conclusion, this study demonstrated a significant association between hematological indices and the GRACE risk score in ACS patients. RDW and MPV, moderately correlated with GRACE risk scores, can provide additional diagnostic information in emergency settings and assist in treatment strategy selection. Assessing these indices during hospitalization may aid in risk stratification for ACS patients. The main advantage of using hematological indices lies in their cost-effectiveness and accessibility in clinical practice.
